# A Discourse on the Causes, Effects, and Preventives of Intemperance, in the Use of Ardent Spirits

**Published:** 1828-05

**Authors:** Daniel Drake


WESTERN JOURNAL

OF THE

MEDICAL & PHYSICAL SCIENCES.

MAY, 1828.

### ESSAYS AND CASES.

---

Art. I.—*A discourse on* Intemperance; *delivered by appointment, at a public meeting of the Agricultural Society of Hamilton County, Ohio, March 1st, 1828, and subsequently pronounced, by request, before a popular audience.* *By* Daniel Drake, M. D.

"The pestilence that walketh in darkness."

[*Concluded from No. I.*]

### Sec. v.—Of the Morbid Effects of Ardent Spirits on the Body and Mind.

We have already seen that alcohol is a poison. Taken undiluted, even in no great quantities, it kills suddenly;—used daily, in smaller doses, diluted, it is a slow poison, and kills at a more distant time,—but with equal certainty. Even a single fit of drunkenness is a disease which may prove fatal; and the frequent recurrence of such an attack, especially in early life, is seldom long sustained. Great injury to health and constitution may, however, be occasioned by the habitual use of ardent spirits, though actual intoxication be studiously avoided. The proper limits of a discourse do not admit of a full account of the various disorders of body and mind that are occasioned by this abuse; but enough may be said, to warn those who are about to yield to temptation, of the diseases they should prepare to encounter.

The disorders of body produced by habitual intemperance, are various in different persons, and at different periods of life.

1. In nearly all, the Stomach, from being the receptacle of the poison, becomes greatly disordered. At an early period the appetite begins to decay, and at last is almost extinguished. The stomach becomes irritable, and vomiting occurs in the morning. At length, a great degree of irritation, and sometimes actual inflammation, takes place in the inner surface of that organ; and, indigestion or dyspepsia, with its loathsome crudities and cankering acids, becomes permanently established.

2. Next to the stomach, the Liver is, perhaps, the greatest sufferer. At first it is strongly excited, and secretes an excessive quantity of bile, which leads the inebriate to say that he is bilious; for which, with admirable facility, he resorts to bitters: thus recklessly feeding the flame with spirit, instead of quenching it with water. Actual inflammation at length supervenes, and is harrassed with a slow fever. In the progress of time, the liver becomes changed in its texture, hardened, and obstructed. It fails to secrete bile, and a dark and muddy Jaundice indicates the approach of death, which is seldom slow in arriving.

3. From sympathy with the stomach and liver, or in some other way, the Lungs become affected. A cough supervenes, with an offensive discharge of phlegm in the morning. If the patient were predisposed to asthma, great difficulty of breathing ensues; or, if inclined to consumption, the fatal malady is hastened in its approaches.

4. The feet swell with a Dropsy, not easily removed, and the abdomen becomes the seat of dangerous watery accumulations. Still oftener, a hopeless dropsical effusion takes place into the chest and about the heart, which labours like a wheel under water. The patient has a distressing cough, is unable to ascend to the second story of his workshop, or climb the lowest hill on his farm; finds himself obliged, at length, to abandon every kind of business, for want of breath; and sits up all night for weeks, lest he should be suffocated by lying down—an event which often happens at a moment when it is not expected by those about him. Many remarkable examples of sudden death, from this cause, might be mentioned. I will recite one, which fell under my own observation in this city.

A man with a family, gradually became a sot; and at length fell into a dropsy of the chest. His career of intemperance extended through several years, during which his perversions of temper, and inattention to business, of course led to occasional jarrings between himself and his wife—an ambitious and worthy woman. Finally, he died suddenly, and without any special warning, about the dawn of day, having risen and dressed himself. I reached the house as he breathed his last, and was in no degree surprised, for I had forseen the termination of his career. But some of his neighbors, being ignorant of what might, rationally, be expected, were in a very different state of mind; and before night, it was insinuated that the wife had committed murder. Thus the intoxication of the husband, which imbittered the existence of the wife for years, might at last have brought upon her the horrible reproach of having caused his death, had no professional man been present to make the explanations necessary to her exculpation.

5. Gout is well known to be the offspring of intemperance; but, from the influence of other causes, it is a rare disease in the United States, especially in the West. Rheumatism is, however, a frequent malady; and, in its chronic forms, is often rendered obstinate by intemperance.

6. All drunkards are liable to Sore Eyes. The disease is sometimes of the lids only: at others, of the whole front of the eye-ball, and attended with an offensive discharge. The young drunkard looks upon this malady with concern. He knows it to be the consequence of his intemperance, and fears that it may betray him to society. To prevent this, he resorts to the aid of *bottle* green glasses; but his sagacity is like that of the owl, which thrusts its head into a cavity of the wall where he is confined;—he blinds his own eyes, but leaves those of his neighbors open to his shame; for by this time, his insobriety is disclosed by many symptoms.

7. Another conspicuous sign of intemperance, is the fiery eruption on the nose and upper part of the cheeks, which, by a desecration of language, has been called *Gutta-rosacea*, or Rose-drop. Actual drunkenness is not necessary to the production of these pimples; nor, indeed, are they exclusively confined to those who drink. They so seldom, however, appear on any others, that the man on whom they begin to sprout forth, may reasonably be suspected.

8. I have known several drunkards, in middle life, affected with Leprosy (*Lepra Græcorum*). The whole surface of their bodies became red and puckered; the skin cracked open, and a thin acrid matter oozed forth, attended with intolerable itching; finally, immense quantities of scales, resembling those of small fish, were daily formed, and fell off in loathsome showers. I have never known a patient restored from this disgusting malady, while he continued to drink.

9. A much commoner consequence of intemperance, is weakness of the muscular system, with tremors. This condition of the inebriate prevails in the morning, when his strength is that of a child, while his tremors are those of old age. He is unfitted for any employment that requires steadiness of nerve, and when he signs his name to promissory notes, given, perhaps, for debts contracted in consequence of his intemperance, the zig-zag lines betray to all attentive observers, that they will, probably, never be discharged.

10. Epileptic convulsions are a more dreadful effect of his intemperate course. They are by no means uncommon, and often prove fatal; though, in many cases, not until they have induced fatuity.

11. Apoplexy is also a drunkard's disease. It occurs in every stage of his melancholy career, but oftenest in old age. It sometimes proves mortal in a single day or hour, or may terminate in palsy of one side of the body; but the latter malady often precedes the former. Thus the individual, when apparently in health, begins to limp; and, without any obvious cause, gradually, loses the use of one leg. Possibly his friends know not the reason; for he may have been a quiet sot, avoiding convivial scenes, and pursuing a regulated course of secret drinking. He resorts to liniments and patent essences; but the seal of death is upon him, and his halting continues to increase, till at length a sudden stroke of apoplexy ends his days, or strikes motionless and senseless one half his body; in which state he may continue to live for years, and to impose on her whom he had sworn to support, and protect, and cherish, the most disgusting servitude,—rewarding her, under the degrading task, with all the outbreakings of a perverted and vicious temper.

12. Intemperance predisposes the body to SPONTANEOUS COMBUSTION. On this point facts have multiplied, until the most incredulous inquirer can scarcely retain his doubts. The bodies of corpulent inebriates, when asleep, have, in several instances, taken fire, by the accidental contact of a burning coal or candle, and all the soft parts have been reduced to ashes, or driven off in clouds of thick smoke. To conceive of the possibility of this revolting catastrophe, we need only recollect the combustible nature of fat, and the still more inflammable quality of ardent spirits, which is composed of the very same materials; and which, being swallowed, daily, in excessive quantities, with reduction of food, may be presumed to alter, to a certain degree, the chemical composition of the body. Meanwhile its vital powers become greatly reduced, and thus render it an easier prey to fire or other external agents.

Lastly. Habitual drinking generates a BAD HABIT OF BODY. which renders the individual liable to violent attacks of disease from slight accidents; and causes various disorders to terminate mortally, which might otherwise be cured. Hence the life insurance of a drunkard, in ordinary health, must always be at a higher premium, than that of the temperate—a fact which is doubtless known to the underwriters. I am aware that drunkards boast of escaping many of the epidemic diseases by which the temperate are assailed. But if this happen at any time, it is because the artificial fever is kept up throughout the epidemic. If they relapse into sobriety, they are more liable to attacks than other people; and when assailed, are far likelier to die. The greater mortality of their diseases, is particularly observable in *young men;* of which I might relate several melancholy examples, but a single instance must suffice. A young man, of this city, addicted to habitual and excessive drinking, received a scratch on one of his feet, which, had he been temperate, would have excited but a slight soreness. With a constitution enfeebled and perverted, however, a gangrenous inflammation speedily ensued, and, to the astonishment of those about him, proved fatal within three days!

I leave this unfinished catalogue of bodily ills, to speak of the darker train of mental disorders, engendered by the demon of intemperance. A fit of drunkenness is a paroxysm of delirium—sometimes infuriated, at others mild and idiotic. A drunken man is, always, either dangerous or ridiculous. His senses deceive him, his passions are tempestuous, his judgment overthrown. His actions are senseless and shameless. If good natured, he is a mawkish and insupportable fool; if ill-tempered, a broilsome and abominable monster; who, in a single hour of uproar and aggression, assails the circle which surrounds him, insults the delicate, tramples upon the weak, grapples with the strong, and at length escapes from the scene of blood and bustle, partly sobered by the retaliating influence of sound blows. It must be a powerful agent that can raise such a tumult; and its daily operation cannot fail to carry disorder into all the faculties and affections of the soul.

An early effect of habitual drinking is IRASCIBILITY OF TEMPER. The patient—for such I must consider him—is easily moved; trifles exasperate him, and when they do not come he seeks them. You know not how to meet his wants or wishes. He discards even his own precedents, and reverses the decisions of each preceding day. Thus he is inconsistent, capricious, discontented and impetuous,—unhappy himself, and the cause of inquietude to his family and friends. This morbid irritability is the soil from which his passions shoot up with the energy of the water hemlock and other rank weeds that overrun our vallies. Anger, hatred and jealousy, not measured by the occasions which excite them, contribute to render him contentious and revengeful; or, if the native bent of his disposition was strongly to good nature, he becomes cowardly, whimsical and silly; and inflicts follies on himself and all who come within his reach. Meanwhile his moral principles suffer impairment—his sense of propriety is vitiated—conscience is dethroned—

and even respect for public opinion is suspended or forgotten. Thus the whole range of moral sentiments is laid in ruins. But the desolation is not limited to his heart—it extends equally to his head. At an early period his senses begin to expeperience injury, and at length his powers of perception are perverted, his observations are made inaccurately, his estimate of the truth becomes erroneous, his memory decays, and his judgment is impaired. In the progress of his intellectual overthrow, his conceptions become disordered, and his imagination excited to an unnatural degree. Strange images arise in his mind, and are believed to be occasioned by external objects, when, in fact, he is almost insensible to all that surrounds him. He occupies himself solely with the creations of his own distempered fancy, which, by this time, afford him abundant employment, for he is now actually mad. At one time he converses with friends who are absent—at another receives mandates from Above; or is enraptured with the sight of imaginary groups of celestial visitants, moving through the air; but in the midst of his bright enchantment, sudden darkness surrounds him, and Satan, with a train of frightful goblins, passes dimly but fearfully before his distempered sight. Terror now arouses him to flight; and, if not confined, he courageously encounters real dangers, in attempting to escape from those which are imaginary. But his flight, of course, avails him nothing; for the frightful images cling to him like the poisoned shirt of Hercules. Exhausted, trembling, and delirious, he is at last taken into custody, and compelled to swallow drugs till his illusions are dispelled. He now returns to reason enfeebled in mind and body—mortified at his own recollections—sunk in the confidence of society, and full of reform. But his passion for drink returns with his strength; and he acts over and over the same ridiculous and melancholy part, till a sudden or violent death, relieves his family from the heaviest burden that fate could lay upon them.

Sec. vi.—Of the Desolating Effects of Intemperance on the Peace and Interests of Society.

Daily observation shows, that in proportion as men become drunkards, they cease to be useful to themselves, to their families, or to society. All, therefore, who fall into habits of intoxication, must be lost to the community if they do not reform. Did the evil stop here it might be borne; it would then be no worse, than if so many individuals had prematurely died—whose places might be filled up by others. But the ills which the drunkard inflicts on himself, and those associated with him, are far from being merely negative. His intemperance often continues for years, during which the injury to his fortune and family, and the inconvenience to society at large, are oftentimes incredible.

When a common labourer becomes a drunkard, his family is soon reduced to the utmost need. The more he drinks the less he works, and the greater are his expenditures. He soon begins to contract debts, and falls into arrears at every tavern, grocery, and still-house in his neighborhood. Meanwhile, he ceases to contribute any thing to the support of his family, and at length becomes entirely dependent on their industry. But all the exertions of his wife and children are often inadequate to the maintenance even of themselves; and the deficiency is made up by purchases on credit. Pay-day too soon arrives, and brings with it the officers of justice, who carry off and sell their scanty stock of household goods. Frost and famine, then, invade the cabin, or the single rented room, where many of the comforts of a quiet existence once abounded, and the charity of the neighborhood is called forth; but this, in time, becomes exhausted, and the entire family is thrown upon the overseers of the poor, to be supported from the public treasury, at the expense of the industrious and temperate.

If the drunkard be a mechanic, he is either indulgent or tyrannical to his apprentices, whom he makes wayward by his government, and corrupts by his example. He forgets his promises, slights his work, disappoints his customers; apologises with falsehood, perhaps recovers their confidence, but subjects them to new disappointments, till, in disgust, they abandon his shop, and he goes to jail.

If a farmer, he becomes a common pest, frequenting all public meetings, which he often disturbs, and inflicting visitations on private families, for the sole purpose of obtaining his favorite beverage. Meantime his farm is neglected and overgrown; his grounds are badly ploughed, and his crops neither planted nor gathered in due season. His fences fall down, and his fields are overrun with domestic animals, who destroy in summer, the provender on which they should subsist in winter. His orchards go unpruned, and degenerate like himself. He wastes his choicest timber, and neglects to plant for those who are to succeed him. His sheds and barracks decay; his slender stock of fodder is rotted by the weather; and his lean and shivering cattle stand exposed to the storm. A neighboring still-house is his market place; and to this are his sons from time to time despatched, to barter a portion of his scanty stores, for that which is to undermine his health, and entail upon them the disgrace of his name, instead of the estate of their ancestors. Well might such a family exclaim with the inspired prophet—

> "Our inheritance is turned to strangers, our houses to aliens."
> "We are orphans and fatherless, our mothers are as widows."

If a merchant, a want of system marks all his affairs. His clerks are undisciplined, his customers treated uncivilly, and his accompts inaccurately or negligently kept. He sells without profit that which should have been kept for a better demand, and purchases that which will never sell. He neglects his business in its current operations, and at a moment when his credit would otherwise suffer, throws himself upon the sympathy of his friends for that, which he most probably will never repay. Unsatisfied with the regular profits of his badly conducted business, he engages, when excited by drink, in wild speculations, draws the credulous into dangerous responsibilities, and, finally, beggars his own family by reciprocating the favor.

If a physician, he reads no more; and soon loses much of what he had once learned. He forgets his professional engagements, and disappoints his patients,—to their great irritation, if not actual injury. Instead of acknowledging the truth, he is tempted to offer unfounded excuses, and thus contracts habits of falsehood. But all this is a trifle compared with other delinquencies. He does not recollect from one visit to another, the symptoms and treatment of his patients; and, therefore, can prosecute no systematic or rational method of cure. He observes clumsily, scrutinizes no deeper than the surface, forms hasty conclusions, and prescribes at random. Like a blind Cyclops, he inflicts heavy blows, but knows not whether they fall on the disease or the patient. He writes his prescriptions, however, "*secundum artem*," if not *secundum scientiam;* and, leaving the event with Nature and Providence, seeks for his conscience the quieting influence of another glass.

If a member of the Bar, he is seldom in his office, which he transfers, incessantly, from one part of the city to another, as if in quest of that business which he could only attract and secure, by remaining where he should be found. His places of habitual resort are the coffee house and billiard room; where he wastes the time and intellectual energy which should be appropriated to the service of his clients; thus he is often unprepared for trial; and, they, non-suited or badly defended, must either sustain injury, or seek other counsel. He sometimes even visits the forum drunk; misconceives the points under investigation; provokes the sneers of his brethren; insults the Court; and beneath its indignant rebuke, sits down, or departs, in disgrace, if not in silence.

Is he a statesman? the halls of legislation are defiled—A judge? the bench is polluted—A minister of the gospel? the sanctuary is profaned!

Thus we might travel through all the occupations of society, and not find one on which this vice does not exert a withering influence. But it is around the fireside, withdrawn from the rude world, *at home*—the green house of society—amid the blended sweets of family affection, where love, and peace, and joy, should forever abide, that intemperance displays its gloomiest desolations.

When a son, in early life, contracts habits of intemperance, his prospects are utterly destroyed. Whether he was designed for the farm, for a trade, or for one of the learned professions, the proud hopes which had supported his father and mother through years of anxiety and toil, give way to disappointment and deep foreboding. In the anguish of their souls, although they watch over him affectionately, they almost wish he was no more; but he generally lives to break the hearts which once beat joyfully in the contemplation of his pure and simple manners, his budding intellect, and youthful aspirations. Mournful, indeed, is the fate of those parents, who are doomed to see an only son, the natural inheritor of their name and fortune, desert the narrow path of temperance and virtue, for the broad and beaten track, by which the drunkard descends to infamy and ruin.

But the drunkenness of a son is a small matter compared with that of a husband and father; for, of all on whom the dreadful effects of this vice can fall, none are so exposed as a wife and children:—the wife especially, for whose situation, when the habit is confirmed, there is, in general, no remedy. She is the *great* sufferer; and has just claims on the sympathy of every friend. Being the first to detect the intemperance of her husband, inquietude seizes upon *her* mind, while their friends are generally ignorant and unsuspicious. In a mingled feeling of love and pride, she conceals her painful discovery, even at the expense of candour and her own approbation. She hopes to reform him, and, therefore, hides his disgrace; but all her efforts, even when skilfully directed, are too often unavailing. He readily joins her in condemning intemperance; but, in his slight indulgencies, can discover nothing that need alarm her. He can restrain his appetite for drink, and will do so, when it is necessary; but must himself be the judge of of that necessity; and, in general, never makes a decision. Meanwhile his propensity increases by indulgence; and at every successive night, it might be said, in the language of a homely but appropriate maxim, that *he is one day older, and two days worse.* The partner of his bosom, now alarmed, essays all her arts to arrest his mad career. She reasons, expostulates, and holds up to his view the scorn of society, and the wrath of heaven: she reminds him of his schemes of early ambition, and exhorts him to renew the race of wealth or glory: she invokes the spirit of their first love, reviews their wedded life, and presses into her service every tender recollection: she presents to him the future, as he once had pictured it, in colors brighter than those which gild the morning clouds; finally, she surrounds him with his own offspring, and bids him look upon the little ones, whom he is shortly to reduce to penury and disgrace. But, too often, no reformation takes place; and she at length feels that he is lost. She must then prepare for the "days of darkness" which are to come. Long scenes of fatigue, anxiety and varied mortification stretch out before her;—she recoils, heart-stricken, from the contemplation, but her path lies through them, and she cannot change it's course. Yet she yields to her destiny with reluctance; for woman's lot is cast upon her marriage, and she cannot give up her hopes of domestic happiness, without a struggle. At length their children come to occupy her whole heart; and, for their support and education, she dooms herself to incessant toil, and every privation. Were she alone, her efforts might be successful; but who does not know, that a drunken father is far worse than none? His whole conduct encourages licentiousness, waste, disobedience and disorder among his children and servants, and thus augments, ten-fold, the cares and difficulties of the mother. Thus, the last bright expectation of former days is blasted, and her spirit is crushed anew; for she now foresees the probable ruin of her children, as well as her husband. They, in fact, grow up in ignorance, in spite of her utmost efforts; and while some of them cling to her in pity, others fall into vice, or wander off; and all are far different in character and fortune from what she once had reason to expect. Meantime most of the early friends of the family discontinue their intercourse, and the few who still visit them, but add to her mortification, by witnessing the degraded manners and conversation of her husband. Their little property disappears, piece after piece; and debts are contracted, until both friendship and charity become exhausted. Thus years of bitterness roll away; and she is finally disenthralled by the welcome, though perhaps violent, death of him, for whose happiness she would once have yielded up her own life.

This long expected event spreads a solemn gratification through society; and the members of his own family can scarcely conceal from themselves, and from each other, how much they are relieved. His old friends reappear in the funeral procession; but it is to contrast, in under tones, his early with his latter days,—not to bewail his death. The rites of a decent burial being performed, his grave is, generally, ever afterwards neglected by all his acquaintances. Should the pride of family erect over it a monument, inscribed with a catalogue of the virtues which he had long ceased to practice, the misrepresentation serves but to preserve the memory of his failings; and those who reared it, are seldom led to its perusal. They even avoid the spot; and no wife and children are seen blending their tears with the first dews of evening upon its tender grass; but thorns and thistles are suffered to spring up with an energy emblematical of his vices, and the serpent makes its home beneath their tangled branches.

### Sec. vii.—Of the means of Curing and Preventing Intemperance.

In all stages of his career, if the drunkard refrain from drinking he of course becomes a sober man; and should no permanent disease have been excited in any organ, he may resume his occupations, and, perhaps, return to the place he had filled in society. But, unfortunately, the habit being once established, he will not, I might almost say cannot, refrain. He repents it is true, but drowns the anguish of his remorse in a fresh libation. His morbid appetite, and the artificial demands of his constitution, are too importunate to be resisted. The flesh triumphs over the spirit; and, as the charmed bird advances, a terrified and trembling victim into the serpent's mouth, so he returns, with horror, to the poison which he knows must at length destroy him. Even in this stage, however, he is, occasionally, cured; either by some alarming event, which carries into his system, both moral and physical, a deep revolution—or, by the associated action of a nauseating medicine, which disgusts him with intoxicating mixtures. But the number thus emancipated is extremely small; and it may admit of doubt whether the successful application of a medicinal agent to the cure of drunkenness would not, on the whole, do more harm than good. Such a medicine, it it is true, might save many families from the destruction, which uncured intemperance, in the father, must eventually bring upon them; but there is some reason to apprehend, that *a belief in the existence of such a cure* would tend to diminish the fear of indulgence, in those who are beginning to feel the propensity. Civilians regard the certainty of punishments, as more efficacious than their severity; and, if we open to him, who is commencing a course of drunkenness, the prospect of being cured, at some future time, by the use of medicine, it can scarcely be denied, that a strong inducement for refraining is abolished.

The true and greatest object is prevention. To this every individual should turn his attention in reference to himself—to this should every wife and sister direct her efforts—to this should all benevolent men dedicate their utmost abilities—and on this should the wisdom and power of our legislative assemblies be exercised. I propose to enumerate some of the modes in which a preventive influence may be exerted; but let us first enquire, for a moment, into the moral nature of intemperance.

It has been ranked with vices and crimes, when it should, rather, have been classed with the bodily disorders which, carrying disturbance into the soul, ultimately lead to their perpetration. This estimate of its character is not at variance with that assigned to it in our Holy Scriptures. The criminality of the drunkard does not consist so much in the outrages which he commits when intoxicated, as in not having governed his appetites, and resisted the establishment in his system of a morbid action, which may destroy the foundations of reason, and poison the fountains of moral feeling. While these remain unpolluted he is a free agent; and is criminal if he do not oppose considerations of duty to the cravings of appetite. He shoul.l consider the danger of exposing himself to temptation, and the immorality of an indulgence, which all experience demonstrates, must, finally, overwhelm both soul and body. Yet when he has yielded to the tyranny of a morbid appetite, and become a sot, he should not be treated with cruelty; for that would neither cure him, nor deter others. On these principles should society rest his case; for, upon them, only, can we construct a rational system of prevention.

The principal elements of this system may be distributed into several groups.

### I. ABOLITIONS.

The daily use, in families, of ardent spirits, whether under the name of bitters, slings, toddies, juleps, punch, or grog, should be discontinued.

The absurd custom of offering them to visitors should be renounced.

The license for drinking establishments, under the denomination of groceries, coffee-houses, and confectionaries, should be granted on the principle of suppressing them, instead of deriving from them, as at present, a revenue that shall diminish our taxes.

The use of whiskey by the engineers, artists and operatives in work-shops and manufactories, should be discouraged.

Farmers of wealth and moral influence should agree with each other, not to distribute that liquor among those whom they employ as labourers in harvest time, and on other special occasions, which are most erroneously supposed to require it.

Finally, the practice of drinking ardent spirits at all sorts of public meetings—a practice which interferes as much with the objects for which they are held, as it corrupts the habits of the people at large—should be denounced and abolished.

### II. SUBSTITUTIONS.

Very great advantage to the interests of temperance would result from substituting several different beverages, of a stimulating quality, for ardent spirits. The inhabitants of wine countries are generally temperate; and the same remark will apply to those which abound in cider. When cider and spruce beer were the national beverages of New England, her farmers and mechanics were patterns of sobriety. Of the change that has followed, upon the profuse distillation of apple-brandy and molasses rum, I shall leave it with her own moralists to speak. Strong beer and porter are preferable to ardent spirits; but their daily use leads to corpulence, apoplexy and sottishness. Table and family beer and porteree are far healthier, and never intoxicate. They cannot stimulate a stomach seared with the products of the distillery; but to any other they impart as much excitement as comports with sound health. Even lemonade and sherbet are beverages which, on many public occasions, might be substituted for strong drink. In Paris the men of letters, assembled in large evening parties, often refresh themselves with lemonade only, while for hours they sustain the highest grade of intellectual excitation. Of cordials I cannot speak in terms of commendation. They abound in alcohol, rather mixed than chemically combined with other ingredients; and, while the latter entice us into excessive drinking, the former produces almost every mischief that can flow from ardent spirits. But of tea and coffee, as substitutes for alcoholic compounds of every kind, an opinion too favourable could scarcely be expressed. I am not unaware of the injuries which these infusions occasion; especially in females and young men of studious and sedentary habits; but drunkenness is not of the number, nor are all the evils which they produce to be compared with that single vice. That their daily use, particularly the use of tea, contributes to the prevention of intemperance, there cannot be a doubt. I have not often seen a tea drinker, become a drunkard; and quite as seldom known a man remain temperate. who preferred an evening draught of spirit and water, to that delightful beverage. Of every known stimulus it seems, indeed, to raise in our faculties and feelings the pleasantest animation; and hence its use by so many millions, inhabiting continents the most remote from one another, may be explained if it cannot be fully approved.

III. LEGAL DISABILITIES AND PENALTIES.

Our statute laws should be so framed, that drunkenness would bring on those who practice it, several disabilities, the anticipation of which might contribute to deter thosewho are about to yield to temptation.

1. The drunkard should not be eligible to any office in our judiciary.

2. When called to serve as a juryman, either party should have the right of challenge for his failing.

3. When subpœnaed as a witness, the party against whom he is to testify, should be permitted to offer proofs to the court and jury, of his habitual intemperance; that they might make the necessary allowances for the mistakes into which he is more liable to fall than sober men.

In all cases the law would proceed on the fact, that habitual intoxication disorders our external senses, and impairs our mental faculties; and that the observations and conclusions of the drunkard, are at no time to be received without distrust of their accuracy. As a witness he might still remain *competent*, and, as it respects his *disposition* to know and state the truth, even *credible;* but his *capability* should be questioned, for it is impaired.

In permitting the habitual drunkard to give testimony, without its being shown to the court and jury that the is such, the law seems to me to be deficient in its usual perspicacity. The man who is frequently intoxicated, ceases at length to be of sound mind; and should be estimated accordingly. When from this cause he falls into manifest derangement, (*Delirium tremens,*) he is no longer competent as a witness; but this state is only the consummation of an intellectual perversion which commenced long before, and, insidiously, deteriorated his powers of perception.

4. The property of the drunkard should be placed in the hands of trustees. If this were done at an early period, it could scarcely fail to contribute to his reformation; and when it did not succeed, both he and his family might, eventually, be kept from becoming public charges. In some of the states, a law of this kind exists; and it should be adopted by the whole.

IV. SOCIAL DISABILITIES AND RESTRICTIONS.

If these were properly and generally imposed, they would contribute signally to the prevention of drunkenness.

No professional man or mechanic should be employed, after he is known to be falling into habits of intemperance.

Boys should not be placed as students, apprentices or clerks, with intemperate men. This restriction is called for, not less by a regard to the interest of the boy, than the reformation of the inebriate.

Capitalists and master workmen of every kind, should refuse to employ, as operatives or journeymen, all who are given to drunkenness.

Other restrictions of a similar kind might be mentioned, but these will serve as specimens.

V. DOMESTIC RESTRICTIONS.

Under this head I would embrace the influence of the sex. If judiciously and earnestly exerted, it would be very great; but I am sorry to say, that in general it is not. Women, both married and single, seldom frown upon intemperance, in its early, which are its only curable, stages,—however they may detest its confirmed and loathsome shapes. Every young woman of sense and virtue, instinctively shrinks from the idea of marrying a man of suspected integrity, or of manners, vulgar in comparison to her own; and why should she not take exception to that which is still more portentous? To that which destroys both honor and refinement, and brings in its train such woes, as neither dishonesty nor rudeness can originate? If the parents and daughter were not to trust a suitor, who has been once convicted of drunkenness, they would do more to promote the temperence of young men, than all the moralists and declaimers of the age.

The young wife, however, rests under the heaviest responsibility. It is she who has the deepest stake in her husband's habits, and may exercise over them the greatest power. She ought, therefore, to study this subject thoroughly; and take her stand, at an early period, in a spirit of mild but dauntless resolution. Her motto should be that of the Roman moralist: *Resist the beginnings.* The *custom* may be broken up, but the *habit* is indestructible. Whatever is done, should be done quickly; but it must be done skilfully. It is necessary that she should retain her husband's affection, while she opposes his propensity to intemperance,—a delicate, but in many cases not an impossible task. In proportion as she discovers an increasing necessity for her interference, she should display a deeper affection,—as her object is not to punish, but to preserve. She must render home more and more attractive to him, and seek to establish with him a closer and dearer companionship. She must not withdraw from her female friends; but, by every practicable means, unite her husband with herself, in the enjoyment of their society; for no man would desire or dare to become a drunkard in the midst of virtuous women. But while she does all this and much more, she ought not, even for a single moment, to forget the object in view; nor so speak or act, as to make her husband suppose her indifferent to his failing. She must never, by word or deed, sanction the daily use of morning drams; nor look, as I am sorry to say, she too often does, with levity on his first frolicksome indulgencies in company. Under every aspect it can assume; by whatever name it may be called; in spite of the most plausible pretexts; and in the face of the most honorable examples to bear him out, she must frown upon every excess. When he comes home, in season, but inebriated, she should receive him with sadness and reserve; and let him, if he choose, revenge himself by returning to the scene of his dissipation: He will at last make his reappearance sober. At other times, when he remains away, she should not retire to rest; but, in a feeling of desperation, watch out the longest nights; that he may be touched by the anguish of her spirit, and dismayed by the firmness with which she has resolved to make no compromise with his failing.

If no amendment take place under this simple method, the case is obstinate; and she should prepare for every thing, *but early and silent acquiescence.* It will be due time for this, when all the means within her reach have been employed without avail. Before that state arrives, her activity should be ceaseless; and her efforts directed with all the sagacity she can summons into her service. She must not be discouraged at the inefficiency of one attempt; but pass to another, trying all things, and holding fast to that which is good. Thus rising in her energies with the growth of his vice, and adapting her means as far as possible, to his peculiarities of temper and disposition, she must, at different times, and under various circumstances, entreat him with exhortations,—confound him with arguments,—alarm him with consequences,—reproach him with injuries,—overwhelm him with the tears of embittered love! Let her not be alarmed at this advice. She has nothing to fear, and something to hope, from a determined course. Her husband, knowing her to be right, and being conscious that he is wrong, will be compelled to respect her in the midst of his irritation; and, while he might turn with contempt from the sighs of weakness, may cower beneath the remonstrances of indignant love. There is power in the stern virtue of a woman's heart; and no husband, not brutal by nature, or from vice, can set at naught her firm resolves in the cause of duty. When pressed to extremity, her reaction has stricken terror into him, who, till then, never felt alarm. Endowed by her Creator with this peculiar power, the sustaining principle of her sex's dignity, it is her duty to exert it. What! is she a bondwoman, or a beast of burden? Is she to cater for his appetites, and waste her days, in servitude to his unhallowed passions? Was it for this she departed from the mansion of her ancestors, and relinquished the endearing protection of her father and brothers? Was it for this she stood before the altar and exchanged vows of fidelity and love?

Is she not rather in many respects a coequal, with rights and dignities not dependent on the will of her husband? If her sphere of action be more limited than his, is she not a free agent within her proper circle, and should she not fearlessly maintain her interests? God has given her a desire for happiness, and the liberty to pursue it according to certain laws; and should her husband's vices obtrude upon her narrow and rugged way, she must effectually dispel them, or relinquish every hope save that of Heaven.

But great as may be the power of woman, when timely and courageously exerted, we must not rely on it alone, to repress or controul intemperance. A variety of causes tend to paralize her efforts, such as ignorance; native timidity; an indifference to remote consequences; the habit of unqualified submission; and, above all, the wide spreading and fatal weakness, of allowing her husband, for a time, to atone for his excesses by redoubled attentions to herself. It is our duty, as men, to look to ourselves and to each other. The practices which, reciprocally, corrupt our lives should be abolished; and in their stead we should adopt those which refine our manners and purify our hearts. We should follow, in reference to vice, the economical maxims, by which we guard against losses by fire and flood. We form mutual insurance companies for our property, why not for the virtues by which that property is to be earned and held? An estate is soon lost, if not guarded by temperance and its associates, industry and economy; and when lost by drunkenness it is gone forever. It is melancholy to witness the folly of poor inconsistent man. To look at his long policies of insurance against the torch of the incendiary, when his property at that very hour belongs of right to other men, who have administered to his intemperance, or profited by the stupidity which it generates. Let us, then, awaken from the sleep of indifference, and open our eyes to the nature and effects of that vice, which, above every other, brings distress and ruin upon the land. There are several causes which contribute either to diminish our aversion to it, or to quiet our consciences under the neglect of the proper means for its suppression.

1. We are accustomed to witness it from infancy, and are, therefore, the less offended by its obscenities, while we grow up with a feint impression, that it is a necessary—or at least an inevitable—evil in society.

2. It connects itself with the private lives and domestic habits of the individuals who practise it; and is thus supposed by many, not to be an admissible object of public censure. But this is an error which ought to be exploded. If the drunkard, sooner or later, require the interposition or the guardianship of society—and this he may do—society has the *right*, if it can devise the means, to *prevent* his drunkenness. He becomes delirious and requires confinement; he abuses or assaults his family, who must seek the protection of friends or of the laws; he wastes his estates, and throws both himself and children on the bounty of the commonwealth:—these are but a part of the evils which he inflicts upon the state, and still they are enough to make him a fit subject of both social and political legislation.

3. No vice in general makes a more clandestine progress. In a few cases it is clamorous, but then comparatively harmless—its deadliest forms are silent, and give little alarm. Its march is unobserved; for it comes not with drums and trumpets, but steals upon society like an armed man under cover of the night, and frequently is not discovered till resistance is of no avail.

4. Many worthy men are deterred from interfering, by their want of moral courage. They fear the sarcasms of the drunkard and his dissolute associates. They alarm themselves and each other, with possible consequences; and while they would delight to witness the reform even of a single inebriate, they are unwilling to risk a solitary effort to accomplish it. They are frightened by the magnitude of the duty into its total dereliction. It would, perhaps, be wrong to press such persons into the army of reform, for Nature has given them a commission of neutrality, and her power should be respected.

5. But the great cause of our remissness lies in the excessive energy of the selfish principle. Its power is sometimes almost magical; for, like an inverted telescope, it presents to us the evils of all other persons in reduced dimensions, and enables us to bear them with the most admirable fortitude. The man of inordinate selfishness is never able to find in the community a single evil, which *duty* calls upon him to correct. The sufferings of other people cannot pierce the coat of mail in which his heart is incased. His partnership with society is not one of profit and loss; but of clear gain, if he can make it so. When dividends are to be declared, he is a large stockholder; but if disasters are to be met, or deficiencies supplied, he is no longer a member of the firm. He looks upon society as something created for his use; and delights in its prosperity, whenever that prosperity will contribute to his benefit; while he deplores the miseries of others, as far as they may detract from his own interests or enjoyments, and no further. By the most touching events connected with others, his heart is not warmed to the core; and the tender charities which begin to sprout up, soon perish by the frost within. Thus the utmost power of the sun, thaws but the surface of the shores around Hudson's Bay; and the young plants which the famishing adventurer would cultivate, are speedily chilled by the ice beneath.

It is edifiying to observe his conduct in particular situations. Thus at the dead hour of night, when the city is alarmed by the cry of fire, and every noble spirit, whether rich or poor, instinctively hurries to the spot; when the sounds of dismay and lamentation reach the deep recesses of every generous heart, and even the tear of pity starts in many an eye, the selfish man repairs to his chamber window, to observe the course of the wind! If the lurid flames are driven towards his own estates, they melt his heart into the deepest sympathy. He then becomes sorely afflicted for the loss of his suffering neighbors, and brings to their relief his mightiest efforts; but let him feel secure from the evil, and he gazes at the conflagration like a second Nero.

From such men, the philanthropist need expect no aid in his schemes of charity; but this should not discourage him; for a daring minority in the cause of virtue, will make itself felt and feared. Ardour and union must produce important results, in spite of every opposition. The true corrective of intemperance is public sentiment; which should every where be embodied and directed with skill and intrepid perseverance.

Let the habitual use of ardent spirit be discountenanced, both by example and precept: let drunkenness in all its grades and varieties be declared infamous: make it an offence against the laws of society, as it is against the laws of Nature and of God: spurn it with contempt: display the list of its murders: proclaim its guilt to the world; and pronounce upon it, a sentence of perpetual banishment.

Thus would the "pestilence that walketh in darknesss" be stayed in its onward course,—the imprudent warned in time of the danger which surrounds them,—and a vast multitude preserved, from the ravages of its destroying power.

